# Do Blood Phenotypes of Feline AB Blood Group System Affect the SARS-CoV-2 Antibody Serostatus in Cats?

**DOI:** 10.3390/v14122691

**Published:** 2022-11-30

**Authors:** Eva Spada, Federica Bruno, Germano Castelli, Fabrizio Vitale, Stefano Reale, Vito Biondi, Antonella Migliazzo, Roberta Perego, Luciana Baggiani, Daniela Proverbio

**Affiliations:** 1 Laboratorio di Ricerca di Medicina Emotrasfusionale Veterinaria (REVLab), Dipartimento di Medicina Veterinaria e Scienze Animali (DIVAS), Università Degli Studi di Milano, 26900 Lodi, Italy; 2 Centro di Referenza Nazionale per le Leishmaniosi (C.Re.Na.L), Istituto Zooprofilattico Sperimentale (IZS) Della Sicilia A. Mirri, 90129 Palermo, Italy; 3 Department of Veterinary Sciences, University of Messina, 98168 Messina, Italy; 4 Dipartimento di Prevenzione, Area Sanità Pubblica Veterinaria, UOC Sanità Animale, Igiene Degli Allevamenti e Produzioni Zootecniche, Asl Latina, 04100 Latina, Italy

**Keywords:** severe acute respiratory syndrome coronavirus, cats, blood phenotypes, risk factors, epidemiology

## Abstract

Cats are susceptible to coronavirus infections, including infection by human severe acute respiratory syndrome coronavirus (SARS-CoV). In human ABO system blood groups, alloantibodies can play a direct role in resistance to infectious diseases. Individuals with the AB blood type were over-represented in the SARS-CoV-2 infection group. Blood type AB individuals lack both anti-A and anti-B antibodies, and therefore lack the protective effect against SARS-CoV-2 infection given by these antibodies. Starting from this knowledge, this pilot preliminary study evaluated a possible association between feline blood phenotypes A, B, and AB and serostatus for SARS-CoV-2 antibodies in cats. We also investigated selected risk or protective factors associated with seropositivity for this coronavirus. A feline population of 215 cats was analysed for AB group system blood phenotypes and antibodies against the nucleocapsid (N-protein) SARS-CoV-2 antigen using a double antigen ELISA. SARS-CoV-2 seropositive samples were confirmed using a surrogate virus neutralization test (sVNT). Origin (stray colony/shelter/owned cat), breed (DSH/non DSH), gender (male/female), reproductive status (neutered/intact), age class (kitten/young adult/mature adult/senior), retroviruses status (seropositive/seronegative), and blood phenotype (A, B, and AB) were evaluated as protective or risk factors for SARS-CoV-2 seropositivity. Seropositivity for antibodies against the SARS-CoV-2 N-protein was recorded in eight cats, but only four of these tested positive with sVNT. Of these four SARS-CoV-2 seropositive cats, three were blood phenotype A and one was phenotype AB. Young adult age (1–6 years), FeLV seropositivity and blood type AB were significantly associated with SARS-CoV-2 seropositivity according to a univariate analysis, but only blood type AB (*p* = 0.0344, OR = 15.4, 95%CI: 1.22–194.39) and FeLV seropositivity (*p* = 0.0444, OR = 13.2, 95%CI: 1.06–163.63) were significant associated risk factors according to a logistic regression. Blood phenotype AB might be associated with seropositivity for SARS-CoV-2 antibodies. This could be due, as in people, to the protective effect of naturally occurring alloantibodies to blood type antigens which are lacking in type AB cats. The results of this pilot study should be considered very preliminary, and we suggest the need for further research to assess this potential relationship.

## 1. Introduction

Blood types are markers on the surface of erythrocytes that can induce an immune response in other individuals with different blood types. Cats have one major blood group system, the AB group system, which consists of three phenotypes, the common type A, the less common type B, and the extremely rare type AB [1,2,3,4]. The form of the neuraminic acid on the red blood cell (RBC) membrane is the major determinant of blood group antigens in cats [5,6]. Disialogangliosides from the membranes of type B RBCs express solely N-acetyl-neuraminic acid (NeuNAc), while type A RBCs have predominantly N-glycolyl-neuraminic acid (NeuNGc)-containing gangliosides and smaller quantities of gangliosides containing NeuNAc. Equal amounts of NeuNAc and NeuNGc disialogangliosides can be recovered from type AB RBCs [7,8]. In addition, cats with some blood types have natural, preformed alloantibodies against blood type erythrocyte antigens that they lack. Cats with blood type A have no, or weak, anti-B alloantibodies, cats with blood type B have strong anti-A alloantibodies, and cats with type AB have no naturally occurring alloantibodies [2,9]. In transfusion medicine the determination of blood type is important to reduce the risk of reaction due to blood type incompatibility and to prevent feline isoerythrolysis in kittens with blood type A born to a blood type B mother [10,11]. 

In humans, blood groups can also play a direct role in susceptibility to infection by microorganisms, parasites, and viruses. Many blood groups are in fact receptors for bacteria and parasites, and these can facilitate the invasion of cells or the evasion of the host’s defense mechanisms [12,13]. In the past decade, many studies have found associations between human ABO blood groups and susceptibility or resistance to coronavirus infection, and, in particular, studies report that blood type O individuals have a significantly lower risk of infection by severe acute respiratory syndrome coronavirus 2 (SARS-CoV-2) [14,15,16,17,18]. Similar associations had previously been reported with SARS-CoV-1 infection during the infection outbreak in early 2003 [19]. 

Coronavirus infection disease 2019 (COVID-19), the disease caused by SARS-CoV-2, a new coronavirus isolated in China in late 2019, rapidly evolved into a global pandemic [20]. Amongst Europe countries, Italy was severely affected, beginning in the second half of February 2020 [21]. Under natural conditions, domestic cats were found to be susceptible to SARS-CoV-2 infection. A number of studies have shown that cats can be infected and develop antibodies to SARS-CoV-2 [22,23,24,25,26,27,28,29,30,31,32,33,34,35,36,37,38,39]. The statistically significant risk factors for infection or seropositivity mainly relate to living in a home with a person infected by SARS-CoV-2 [24,30,31,36,37,38,39] and, in particular, cats that are in close contact with infected owners are at a higher risk of becoming infected than other, unexposed animals [28,36]. Sharing a bed with the infected owner or being kissed by owners were significant associated risk factors for pet infection [30,38]. In addition, neutering status was also associated with infection [38], as well as resulted SARS-CoV-2 seropositive status and the onset of clinical respiratory, gastrointestinal, or systemic signs such as lethargy [30]. The source of the cat could also represent a risk factor for SARS-CoV-2 seropositivity. In fact, compared with cats originating from households, cats that were in a shelter, rescue, or foster facility were 3.6 times more likely to be seropositive [40]. Studies had assessed but not found significant associations between SARS-CoV-2 status in cats and age, sex [25,29,30,34,36,38,41], breed [25], contact with the outdoors [41], number of cats in the household [24,30,31], and coinfections [29,38].

Based on the penetration of the SARS-CoV-2 infection into feline populations, and with knowledge of the association between human ABO blood types and susceptibility to SARS-CoV infection, we performed this preliminary pilot observational study to evaluate a possible association between feline blood phenotypes A, B, and AB and SARS-CoV-2 antibody serostatus. The second aim of this study was to investigate the risk or protective factors associated with seropositivity for SARS-CoV-2 in cats.

## 2. Materials and Methods

This preliminary observational study was performed between April 2021 and March 2022 in stray colony, shelter, and owned cats from different provinces in Lombardy, northern Italy, evaluated for routine check-ups before neutering or for diagnostic purposes in the case of sick cats (owned cats), prior to adoption or neutering (shelter cats), and before neutering and for preventative health reasons (stray colony cats). In shelter and stray colony cats, oropharyngeal and rectal swab specimens were also collected when the cats were under general anesthesia for neutering surgery.

The following data were registered for most cat: origin (stray colony, shelter, or owned cats), breed (domestic shorthair—DSH or purebred cats), gender (male or female), reproductive status (intact or neutered), age (categorized as kitten, from birth to 1 year; young adult, from 1 to 6 years; mature adult, from 7 to 10 years; and senior, more than 10 years) [42]. 

The protocols for the study and animal welfare were reviewed and approved by the Animal Welfare Bioethical Committee of the University of Milan (approval number OPBA _34_2021, released on 12 March 2021). 

### 2.1. Blood Typing and Back Typing

**Figure 1:**
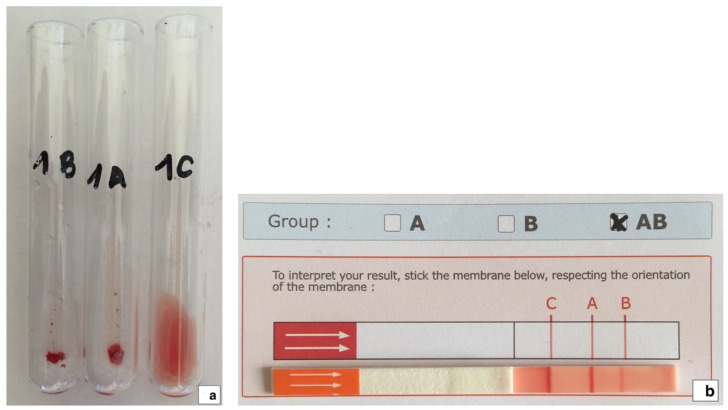
(**a**). Feline blood sample shown to be phenotype AB using the agglutination on tube technique. (**b**) The same sample was confirmed as phenotype AB using an immunochromatographic test (Lab test A + B feline, Alvedia, Limonest, France).

AB blood group system blood typing was performed at the Veterinary Transfusion Research Laboratory (REVLab), Department of Veterinary Medicine and Animal Sciences (DIVAS), University of Milan, Italy, using the agglutination on tube technique (Figure 1a), as previously described [2,43]. All samples identified as phenotypes B and AB with the tube technique were confirmed using an immunochromatographic test (Figure 1b) and the back typing technique, as previously described [2,43,44]. 

### 2.2. SARS-CoV-2 RNA Extraction and Real Time Reverse Transcription Polymerase Chain Reaction (rRT-PCR)

SARS-CoV-2 RNA was extracted from oropharyngeal and rectal swab specimens using the Mag-Bind^®^ Viral RNA Xpress Kit (Omega Bio Tek, Norcross, GA, USA) according to the manufacturer’s instructions. The samples were tested for SARS-CoV-2 RNA in assay RT-PCR to check for the presence of E, N2, and RdRp gene sequences (TIB Molbiol Lightmix^®^ Modular Assays, Berlin, Germany). qPCR reactions were carried out using an Applied Biosystems QuantStudio 3 PCR System (Thermo Fisher Scientific, Waltham, MA, USA). All the procedures were performed as previously described [35].

### 2.3. SARS-CoV-2 Serological Testing

**Figure 2:**
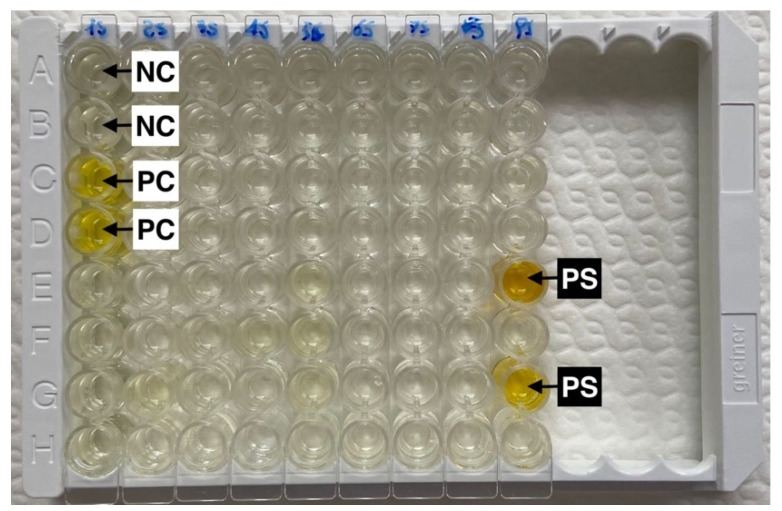
Double antigen ELISA that detects specific anti-SARS-CoV-2 nucleocapsid (N-Protein) antibodies (ID-Screen^®^ ELISA, SARS-CoV-2 double antigen multi-species, ID.vet, Grabels, France). Two positive cat serum samples (PS, positive sample) show a sample/positive control (PC) ratio greater than 60%. The negative serum sample controls are indicated as NC (negative control).

The presence of antibodies against the SARS-CoV-2 antigen was tested using a double antigen ELISA (ID-Screen^®^ ELISA, SARS-CoV-2 double antigen multi-species, ID.vet, France), following the manufacturer’s instructions, and as previously described [35] and used in other studies [25,26,36]. This kit detects specific anti-SARS-CoV-2 nucleocapsid (N-Protein) antibodies. In each sample evaluated, the “Sample/Positive control” (S/P) ratio was calculated and expressed as the S/P percentage (S/P%). Based on the manufacturer’s cut-off, samples with an S/P% equal to or greater than 60% were considered positive, those between 50% and 60% were considered doubtful, and those equal to or less than 50% were considered negative (Figure 2).

Samples positive for antibodies against the N-protein of SARS-CoV-2 were analyzed using a surrogate virus neutralization (sVNT) test (The GenScript cPass™ SARS-CoV-2 Neutralization Antibody Detection Kit, Gen-Script Inc., Piscataway, NJ, USA). This sVNT detects antibodies that block the binding of the SARS CoV-2 receptor binding domain (RBD) to the angiotensin-converting enzyme 2 (ACE2) receptor on a cell surface according to the neutralizing antibodies present in the sample, and it has previously been validated for the detection of SARS-CoV-2 antibodies in cats [45]. This was performed in an external laboratory which had no knowledge of the SARS-CoV-2 status of the samples submitted. Samples with a percentage of inhibition value > 30% were considered positive for the presence of SARS-CoV-2 antibodies. 

### 2.4. Additional Analysis

All samples were simultaneously tested for the presence of antibodies to FIV target antigens p24 and gp40, and the FeLV p27 antigen, using plasma, serum, or whole blood samples and a commercial rapid ELISA kit (SNAP^®^ Combo Plus FeLV Ag/FIV Ab, IDEXX Laboratories, Europe, Westbrook, ME, USA).

Samples positive for the antibodies against the N-protein of SARS-CoV-2 were also tested by an external laboratory with an indirect semiquantitative immunofluorescence (IFAT) detection for specific IgG antibodies against feline coronavirus (FCoV) (Mega FLUO FCoV, MEGACOR Diagnostik GmbH, Gemeinde Hörbranz, Austria). 

### 2.5. Statistical Analysis

Statistical analyses were conducted using commercially available software (MedCalc^®^ Statistical Software version 20.115, MedCalc Software Ltd., Ostend, Belgium). Descriptive statistics were used to summarize demographic and blood type results. The distribution of numerical data (age in years and months, ELISA “Sample/Positive control” (S/P) ratio, and sVNT% of inhibition) was assessed using a d’Agostino–Pearson test and described as mean, range, and standard deviation (SD) if normally distributed or as median, range, and 25–75% percentile if not normal distribution. Associations between factors of origin (stray colony/shelter/owned cat), breed (DSH/non DSH), gender (male/female), neutering status (neutered/intact), age class (kitten/young adult/mature adult/senior), retroviruses serostatus (FIV seropositive/negative, FeLV seropositive/negative), blood type (type A, B, or AB), and SARS-CoV-2 serological status were investigated using a chi-square (χ^2^) analysis or Fisher’s exact test as appropriate. Factors associated with seropositivity from the univariate analysis were tested with logistic regression. A Hosmer–Lemeshow goodness of fit test was used to test for the model diagnostics. Results were considered significant when *p* < 0.05. Odds Ratios (OR) with a 95% confidence interval (CI) were calculated for factors for which a significant association was identified.

## 3. Results

The surveyed feline population was composed of 215 cats, of which 109 (50.7%) were stray colony cats, 67 (31.2%) were owned cats, and 39 (18.1%) were shelter cats. They were mainly domestic shorthair (DSH) cats (n = 193, 89.8%), 52.6% were female and 47.4% were male cats. They were mostly intact cats (64.2%) and young adults (41.4%) (Table 1). Age was recorded for 202 cats, with a median of 2 years (range 3 months–20 years, 25–75% percentiles: 1–6 years). Out of the 213 cats tested, 8 (3.8%) were FeLV seropositive, 22 (10.3%) were FIV seropositive, and 5 (2.3%) were FeLV and FIV coinfected. 

Blood type A was identified in 193 cats (89.7%), type B in 15 cats (7.0%), and type AB in 7 cats (3.3%). All type B and AB samples were confirmed by immunochromatographic and back-typing techniques. 

Seropositivity for antibodies against the SARS-CoV-2 N-protein was recorded in eight cats (3.7%) with S/P% values ranging from 88% to 422% (mean 209%, SD ± 132%).

**Figure 3:**
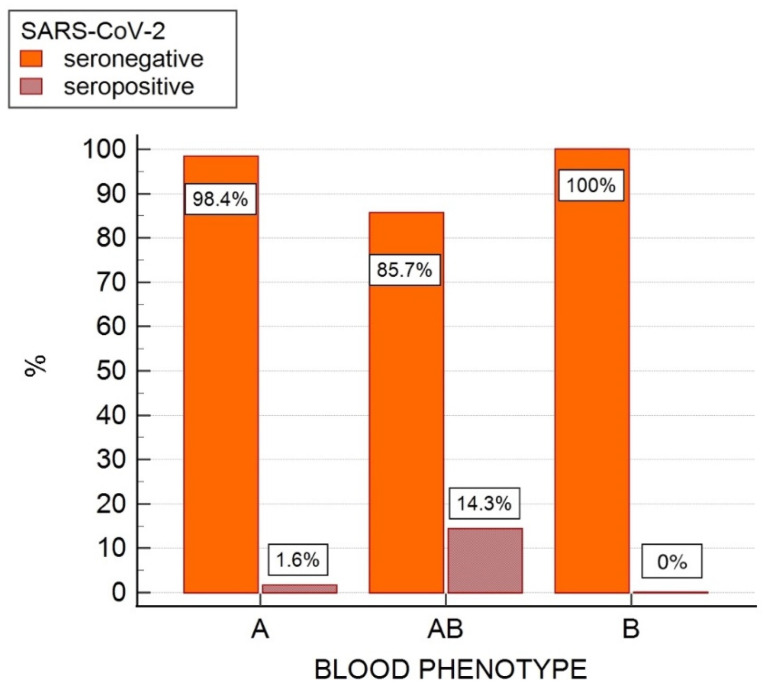
Frequency bar chart showing AB blood group system blood type prevalence (%) in SARS-CoV-2 seropositive and seronegative cats in a feline population of 215 cats from northern Italy evaluated for an association between blood phenotype and serostatus for SARS-CoV-2 antibodies.

Using sVNT test for the detection of SARS-CoV-2 antibodies, four samples positive for antibodies against the SARS-CoV-2 N-protein were found to be seronegative with an inhibition of <30%. Therefore, SARS-CoV-2 seropositivity was confirmed in only four cats (1.9%) with inhibition values ranging from 57% to 89% (mean 82%, SD ± 14%). Of these four SARS-CoV-2 seropositive cats, three were blood type A and one was type AB, while no type B cats were found to be SARS-CoV-2 seropositive (Figure 3). 

All 148 oropharyngeal and rectal swabs collected from the shelter and stray colony cats tested negative for SARS-CoV-2 RNA.

The demographic, historical, clinical, and laboratory data of cats seropositive in all tests for antibodies against SARS-CoV-2 are summarized in Table 2. 

The results of the univariate analysis of factors and seropositivity for SARS-CoV-2 are reported in Table 1. Young adult age class (*p* = 0.0220), FeLV seropositivity (*p* = 0.0244), and blood type AB (*p* = 0.0136) were significantly associated with SARS-CoV-2 seropositivity according to the univariate analysis. Logistic regression confirmed only blood type AB (*p* = 0.0344, OR = 15.4, 95%CI: 1.22–194.39) and FeLV seropositivity (*p* = 0.0444, OR = 13.2, 95%CI: 1.06–163.63) as factors significantly associated with SARS-CoV-2 seropositivity.

Out of eight samples seropositive for antibodies against the SARS-CoV-2 N-protein, five were positive on IFAT for FCoV IgG antibodies (62.5%) and three were negative (37.5%) with an antibody titer ranging from 1:100 to 1:400 (Table 2).

## 4. Discussion

While there is growing evidence in human beings that blood groups affect host susceptibility to infections [12,13,46], little is known about this effect in veterinary medicine [47,48]. An association between blood type and infectious diseases in cats has not yet been demonstrated [49,50]. In addition, our understanding of feline blood types is incomplete. The recent identification of five novel feline erythrocyte antigens not belonging the AB blood group system in cats [51] underlines how little we still know about feline blood types.

The surface erythrocyte antigens that determine blood group could influence the resistance or susceptibility of people to many infectious diseases [12,13]. In people, blood group O is characterized by a complete absence of A or B antigens [52]. When exposed to bacteria of the microbiota with glycan patterns similar to either blood group A or B antigens, people with blood group O develop antibodies against A and B antigens, whilst individuals with blood group A or B develop either anti-B or anti-A antibodies, respectively [53]. Only people with blood group AB do not develop such antibodies. In addition, ABO antigens are also expressed on many other cell types, including epithelial and vascular endothelial cells in many organs [54]. The primary target organ of SARS-CoV-1 and SARS-CoV-2 in humans is the lung, and both viruses use angiotensin converting enzyme 2 (ACE2) receptors to gain access to cells [55]. Antigens of blood types A and B are expressed in lung epithelial cells, where viral particles are produced. When produced in cells that express the A or B blood group enzymes, SARS-CoV-1 S glycoprotein virions express A or B antigens according to the patient’s ABO phenotype [56]. Naturally occurring anti-A or anti-B antibodies in blood group O, B, and A individuals bind the coronavirus S protein and block its interaction with ACE2, thereby preventing infection by blocking virus attachment and entry [57,58]. In addition, natural antibodies against blood type antigens could opsonize viral particles leading to complement-mediated neutralization [54]. All these mechanisms may have contributed to the protection of individuals with blood group O during the SARS outbreak. Individuals with blood type AB were over-represented in the SARS-CoV-2 infection group, followed by type A and/or type B individuals, whilst those with blood type O were at the lowest risk of infection [15,56,57,59]. Blood type AB individuals lack both anti-A and anti-B antibodies; therefore, they completely lack the protective effect given by these antibodies. 

The feline AB erythrocyte blood group system antigens are biochemically unrelated to the human ABO antigens [1,5]. The only similarity between the human and feline blood group systems is that cats, like people, have naturally preformed alloantibodies against the blood type erythrocyte antigens they lack, with the exception of type AB cats, which have no naturally occurring alloantibodies as they have both A and B antigens on the surface of their erythrocytes [9]. Even with a small feline population, as in this study, we hypothesize that this characteristic could explain the results of our study, in which the cats with the very rare blood phenotype AB had an increased risk for SARS-CoV-2 seropositivity, as they lack the potentially protective activity of the alloantibodies against human coronavirus infection. Interestingly, this could also explain why, even if not statistically significant, no phenotype B cats tested seropositive for SARS-CoV-2. While cats with phenotype AB blood have no naturally occurring alloantibodies against the blood type antigen they lack [7], all cats with blood phenotype B have high levels of naturally occurring anti-A alloantibodies [1,2,9].

With regard to seroprevalence of SARS-CoV-2, this study is a continuation of our previous epidemiological study on antibodies to SARS-CoV-2, in which only one (1.0%) seropositive sample was detected from tests on 105 stray colony and shelter cats from Monza Brianza province, a suburb of Milan in northern Italy, during the first year of the COVID-19 pandemic in Italy [35]. In this, our second epidemiological survey regarding the second year of the COVID-19 pandemic, the increase in seroprevalence between the two studies, from 1.0% to 1.9%, may be due to the different populations investigated. This study also included owned cats, and two of the SARS-CoV-2 seropositive cats were owned cats. Owned cats may have had closer contact with a person from whom they might have acquired their SARS-CoV-2 infection. Risk factors for SARS-CoV-2 infection or seropositivity are mainly related to sharing a home with a person with a SARS-CoV-2 infection [24] or having closer contact with a SARS-CoV-2 infected owner [30,38,39]. However, in the present study, cats were included without information on their potential exposure to SARS-CoV-2 or information on owner’s disease status, as this was not collected. Therefore, this is only a hypothesis that may explain how the owned cats acquired their SARS-CoV-2 infections. FeLV seropositivity was a risk factor for SARS-CoV-2 seropositivity in the surveyed feline population. FeLV is an oncoretrovirus that is widespread in domestic cat populations and induces both immunosuppressive and neoplastic diseases of the feline lymphoreticular system [60]. Previous studies have examined the prevalence of SARS-CoV-2 and co-infections, reporting no significant correlation between seropositivity and infections with the retroviruses FIV and FeLV [29,38], while prolonged SARS-CoV-2 shedding was observed in a cat that tested positive for FIV [61].

The surveyed feline population was screened for antibodies against SARS-CoV-2 using a widely accessible and easy-to-perform commercial assay, a double antigen multispecies nucleocapsid-based ELISA, and without using a VNT, considered the reference test for SARS-CoV-2 antibody detection in pets [62]. A VNT was not feasible because its execution requires specialized laboratories with containment level 3 facilities, since the isolation of SARS-CoV-2 poses a risk to laboratory staff and therefore requires specially trained personnel. To confirm the double antigen nucleocapsid-based ELISA results, we tested them using an sVNT, which detected neutralizing antibodies. The sVNTs showed high sensitivity and specificity in comparison with the VNT assay, without cross-reactivity with other animal coronaviruses, such as feline coronavirus (FCoV) [45,62]. A recent study which evaluated the diagnostic performance of two different commercially available ELISA immunoassays and an sVNT for the detection of SARS-CoV-2 antibodies in pets found that the overall sensitivity was higher for the sVNT (100%) compared with the nucleocapsid-based double antigen ELISA assays (23%) when compared with results of a VNT. The specificity was 100% for the sVNT, whereas the nucleocapsid-based double antigen ELISA assays showed a 99% specificity [63]. However, we found that half of the ELISA seropositive samples tested negative for SARS-CoV-2 antibodies when tested using an sVNT, highlighting a poor apparent specificity in our nucleocapsid-based double antigen ELISA test. This finding might be explained by a possible cross-reaction with other coronaviruses, and, in particular, with FCoV. In fact, five out of the eight samples positive for antibodies to the SARS-CoV-2 N-protein were also positive for specific FCoV IgG antibodies. Although many studies [33,35,63] have shown no cross-reactivity between antibodies against SARS-CoV-2 and FCoV, a previous study found that several feline serum samples collected before the COVID-19 pandemic were positive for the N-protein when tested using an ELISA, and this was most likely due to antigenic cross-reactivity between SARS-CoV-2 and FCoV type I N-proteins [64]. In addition, that same study found a poor correlation between N-protein ELISA results and the VNT. Several of the pre-COVID-19 samples did, in fact, test positive for the N-protein using an ELISA, and the authors hypothesized that this was due to antigenic cross-reactivity between SARS-CoV-2 and FCoV type I N-proteins [64]. However, no cross-reaction was detected for both N-based ELISA tests with the feline-species coronavirus in a subsequent study [65] since several false positive sera gave negative ELISA test results for FCoV in cats. The authors of that study suspected that other coronaviruses, not yet identified, may also be involved in the non-specific results obtained for the N-protein. Taken together, the results of these previous studies underline that the N-protein does not appear to be a good target for anti-SARS-CoV-2 antibody detection in cats (or dogs). For this reason, we tested our positive samples with an sVNT, which is an effective method for predicting serum neutralization antibodies in cats [63], showing high sensitivity and specificity in comparison with the VNT assay, without cross-reactivity to other animal coronaviruses, such as FCoV [45,62]. 

Our data confirmed that the proportion of the three AB system blood phenotypes in the populations in this study (89.7% type A, 7.0% type B, and 3.3% type AB) was similar to the feline blood group distributions reported in cats in the same area [2,3]. In all European feline breeds, the A blood type is most common, with 76% prevalence, followed by 21% for type B and 3% for type AB [4]. However, when data for only European DSH, the most prevalent breed in our population, are considered, the prevalence of type A, B, and AB changes, with a lower prevalence of type B cats, as shown here and in our previous study on DSH cats in northern Italy [2,3].

This study has a number of limitations. The first is that it is an observational study, and therefore there is always the possibility that uncontrolled confounding factors are impacting the results. In particular, the numbers of type B and AB cats are low in our population, and no attempt was made to increase the natural prevalence of these rare blood types which reflected the prevalence of the AB blood group system blood types reported in previous studies performed in the same area [2,66]. In addition, the low number of cats with neutralizing antibodies, due to the sporadic frequency of SARS-CoV-2 infection among cats, may have limited the significance of the results of this study, potentially increasing the chance of type II error. However, this should be considered a preliminary and exploratory pilot study on the possible effect of blood type on SARS-CoV-2 serostatus in cats, based on knowledge of what has been described in human medicine. In addition, no attempt was made to ensure that the tested individuals were unrelated. However, a parental relationship is very improbable in our cats, as the SARS-CoV-2 seropositive cats came from different feline populations. Finally, we did not evaluate the clinical impact of seropositivity on cats, although in people ABO blood groups also affect the severity of COVID-19 [15,17,67]. However, the detection of antibodies against SARS-CoV-2 does not correlate with COVID-19 in cats which, usually results in mild transient disease with no significant clinical signs or laboratory abnormalities [25,27,37,38]. In fact, three out the four SARS-CoV-2 seropositive cats in our survey had shown no signs of disease. 

## 5. Conclusions

In conclusion, based on this preliminary survey, blood phenotype AB might predispose cats to seropositivity for SARS-CoV-2 infection, and therefore for COVID-19. This could be due, as described in people, to the protective effect against SARS-CoV-2 infection of natural antibodies to blood type antigens that are lacking in phenotype AB cats and that are high in phenotype B cats. These results should be considered very preliminary, and we suggest the need for further research to assess this potential relationship. 

## Figures and Tables

**Table 1:** Characteristics of an Italian feline population of 215 cats investigated for association between blood phenotype and antibodies against severe acute respiratory syndrome coronavirus 2 (SARS-CoV-2) and analysis for factors associated with seropositivity according to univariate analysis with chi square test or Fisher’s exact test.

Parameter (n = Number of Samples Tested)	Variable	SARS-CoV-2 (n = 215)		*p*-Value
		Seropositive (n = 4) n (%)	Seronegative (n = 211) n (%)	
Origin n = 215	Stray colony cats	1 (0.5%)	108 (50.2%)	0.2960
	Shelter cats	1 (0.5%)	38 (17.7%)	0.7199
	Owned cats	2 (0.9%)	65 (30.2%)	0.4127
Breed n = 215	DSH	3 (1.4%)	190 (88.4%)	0.3264
	non DSH	1 (0.5%)	21 (9.7%)	
Gender n = 214	Male	3 (1.4%)	98 (45.8%)	0.2619
	Female	1 (0.5%)	112 (52.3%)	
Reproductive status n = 214	Neutered	3 (1.4%)	73 (34.1%)	0.0965
	Intact	1 (0.5%)	137 (64.0%)	
Age class n = 205	Kitten (0–1 yr)	0 (0.0%)	69 (33.7%)	0.1512
	Young adult (1–6 yrs)	4 (2.0%)	85 (41.5%)	0.0220
	Mature adult (7–10 yrs)	0 (0.0%)	17 (8.3%)	0.5446
	Senior (>11 yrs)	0 (0.0%)	30 (14.6%)	0.4041
FeLV serostatus n = 213	Positive	1 (0.5%)	7 (3.3%)	0.0244
	Negative	3 (1.4%)	202 (94.8%)	
FIV serostatus n = 213	Positive	0 (0.0%)	22 (10.3%)	1.000 (Fisher’s exact test)
	Negative	4 (1.9%)	187 (87.8%)	
FeLV + FIV coinfection n = 213	Positive	0 (0.0%)	5 (2.3%)	1.000 (Fisher’s exact test)
	Negative	4 (1.9%)	204 (95.8%)	
Blood phenotype n = 215	Type A	3 (1.4%)	190 (88.4%)	0.3264
	Type B	0 (0.0%)	15 (7.0%)	1.000 (Fisher’s exact test)
	Type AB	1 (0.5%)	6 (2.8%)	0.0136

SARS-CoV-2: severe acute respiratory syndrome coronavirus 2; DSH: domestic shorthair; yr: year; yrs: years; FIV: feline immunodeficiency virus; FeLV: feline leukemia virus.

**Table 2:** Demographic, history, clinical, and laboratory data of SARS-CoV-2 seropositive cats evaluated for the effect of blood phenotypes A, B, and AB on SARS-CoV-2 serostatus. Cats definitively SARS-CoV-2 seropositive as per positive sVNT results are in bold.

No.	Origin	Gender and Neutering Status	Breed	Age (Years, Months)	Reason for Evaluation	Clinical Status	Retroviruses Status	N-Protein SARS-CoV-2 ELISA (S/P Cut-Off > 60%)	sVNT (Inhibition Cut-Off > 30%)	SARS-CoV-2 rRT-PCR (Oropharyngeal and Rectalswabs)	IgG Antibodies FCoV IFAT (Titre Cut-Off ≥ 1:100)	Blood Phenotype
**1**	**Owned**	**Neutered male**	**DSH**	**6**	**Bite wound**	**Healthy**	**Negative**	**320%**	**88%**	**Negative**	**<1:100**	**AB**
**2**	**Stray colony**	**Intact male**	**DSH**	**3**	**Orchiectomy**	**Healthy**	**FeLV+**	**107%**	**57%**	**Negative**	**<1:100**	**A**
**3**	Owned	Neutered male	DSH	3	FIV infection- related signs	Unhealthy (respiratory and gastroenteric signs)	FIV+	126%	<0.1%	Not done	1:100	AB
**4**	**Owned**	**Neutered male**	**Bengal**	**1**	**Chronic** **diarrhea**	**Unhealthy**	**Negative**	**216%**	**86%**	**Not done**	**1:100**	**A**
**5**	Stray colony	Intact female	DSH	2	Ovariectomy	Healthy	Negative	88%	<0.1%	Negative	<1:100	A
**6**	Stray colony	Intact female	DSH	1.9	Orchiectomy	Healthy	Negative	209%	<0.1%	Negative	1:200	A
**7**	**Shelter**	**Neutered female**	**DSH**	**1.5**	**Retroviruses test before adoption**	**Healthy**	**Negative**	**110%**	**77%**	**Negative**	**1:100**	**A**
**8**	Shelter	Neutered female	DSH	14	Geriatric check-up	Healthy	Negative	422%	<0.1%	Negative	1:400	A

DSH: domestic shorthair; ELISA: enzyme-linked immunosorbent assay; sVNT: surrogate virus neutralization test; rRT-PCR: reverse real time polymerase chain reaction; SARS-CoV-2: severe acute respiratory syndrome coronavirus 2; FIV: feline immunodeficiency virus; FeLV: feline leukemia virus.

## Data Availability

Data supporting the conclusions of this article are included in the report. Raw data are available on request from the corresponding authors.
